# Severe Disseminated Varicella Zoster in an Immunocompetent Adolescent: A Case Report

**DOI:** 10.1177/11795565261447102

**Published:** 2026-04-30

**Authors:** Kelan Queenan, Ronald Williams

**Affiliations:** 1 Penn State College of Medicine, Penn State Milton S. Hershey Medical Center, Hershey, PA, USA; 2 Department of Pediatrics, Penn State Milton S. Hershey Medical Center, Hershey, PA, USA

**Keywords:** infectious diseases, dermatology, otolaryngology, allergy and immunology, anesthesia/pain, neurology, herpes zoster, varicella zoster virus, shingles

## Abstract

We present the case of an immunocompetent, fully vaccinated 17-year-old female who developed severe, disseminated varicella-zoster virus (VZV) reactivation with visceral involvement, Ramsay Hunt Syndrome, and complications of suspected superimposed bacterial cellulitis, chronic pain, as well as postherpetic neuralgia. We aim to highlight the importance of early recognition and multidisciplinary management of varicella-zoster virus in immunocompetent patients to minimize disease complications. All data were obtained from the electronic medical record with permission from the patient and her parent. The patient underwent serial dermatologic examinations and a comprehensive immunologic workup. Positive VZV PCR testing of the lesion and blood confirmed active VZV infection. Despite an intensive multi-drug antiviral regimen, she developed suspected clinical drug resistance—although resistance testing was not performed—progressing to disseminated varicella with hepatic involvement. Inpatient complications included Ramsay Hunt Syndrome, suspected superimposed bacterial cellulitis, opioid withdrawal, and chronic pain. While outpatient, she developed postherpetic neuralgia and psychosocial impairment, preventing her from completing her academic year. This case illustrates a rare, but severe presentation of disseminated varicella-zoster virus with visceral involvement and concomitant Ramsay Hunt Syndrome in a healthy adolescent. It underscores the importance of considering VZV in the differential diagnosis of pediatric rash and neuropathy in all patients, irrespective of immune status.

## Introduction

Varicella-zoster virus (VZV) is a human herpesvirus that causes varicella (chickenpox) and herpes zoster (shingles). Varicella commonly occurs in childhood and presents as a febrile illness marked with pruritic, vesicular skin lesions.^1^ Following primary infection, the virus remains latent in the dorsal root ganglia of the peripheral nervous system. It can then reactivate and cause herpes zoster (HZ), characterized by a burning, painful, vesicular rash localized to a single dermatome.^2^ Herpes zoster typically occurs when there is a weakening of the body’s cell-mediated immune response, such as in elderly individuals or patients who are immunocompromised.^3,4^ VZV reactivation causes significant morbidity, but rarely mortality.^3^

Primary or reactivated varicella-zoster virus may present as a disseminated disease with visceral involvement including pneumonitis, hepatitis, and encephalitis, and potential multi-organ failure.^3^ Disseminated VZV almost exclusively affects immunocompromised patients such as individuals with human immunodeficiency virus (HIV), acquired immunodeficiency syndrome (AIDS), malignancies, or those receiving immunosuppression or high-dose corticosteroid therapy for organ or hematopoietic stem cell transplants.^1,3^ Herpes zoster classically presents with fewer than 20 vesicles in one or two adjacent dermatomes. When lesions affect more than one dermatome, it is termed multi-dermatomal zoster.^1^ We present a rare case of an immunocompetent pediatric patient with a childhood history of VZV who developed reactivated, multi-dermatomal, disseminated herpes zoster infection complicated by Ramsay Hunt Syndrome, visceral involvement, suspected superimposed bacterial cellulitis, opioid withdrawal, postherpetic neuralgia (PHN), and chronic pain.

## Case

A 17-year-old Caucasian female with no past medical history and no previous steroid use presented to the emergency department with a 6-day history of progressive, erythematous, vesicular rash, accompanied by neck pain and stiffness. She denied similar prior symptoms and initially attributed the rash to a tick bite, though no tick or punctate lesion was identified. She reported primary varicella infection at 6 months of age following contact from an infected sibling, although no confirmatory testing was performed at the time. She received all her childhood immunizations in the United States, including 2 doses of the varicella vaccine at ages 12 months and 5 years old.

She was initially evaluated at an outside hospital 2 days prior where she was diagnosed with herpes zoster and was prescribed oral valacyclovir (dose and frequency unknown), lidocaine, and oxycodone. Despite treatment, new vesicles appeared, and existing lesions worsened. Two days later, she developed a unilateral facial droop, hearing loss, tinnitus, and blurry vision, prompting presentation to the emergency department.

On admission, her vital signs were within normal limits. Physical examination revealed a female of stated age in moderate distress due to neck pain. Her head was normocephalic and atraumatic. Her pupils were equally round and reactive to light, and extraocular movements were intact. No relative afferent pupillary defect was noted. There was normal external ear anatomy with right ear erythema and several 1 to 2 mm painful and pruritic vesicles with crusting on an erythematous base along the helix. No lesions were present in the concha bowl or external acoustic meatus. The tympanic membranes were intact without effusion. Weber test lateralized to the left with air conduction greater than bone bilaterally. Cardiac exam revealed a regular rate and rhythm, with a soft, low-pitched, I/VI systolic murmur at the left upper sternal border. No rubs or gallops were present. The lungs were clear to auscultation bilaterally without wheezes, rales, or rhonchi. On neurologic examination, she was alert and oriented to person, place, and time. There was hypoesthesia along the right V2, V3, C3, and C4 sensory dermatomes, while the motor function of cranial nerve V and the remaining cranial nerves II-VI and IX-XII were intact. She had mild facial drooping along the right nasolabial fold and asymmetric smile. However, eyebrow lift was normal bilaterally. Unilateral hearing loss to finger rub and tinnitus were also present. Dermatologic exam demonstrated multiple grouped, ruptured, pruritic, and painful vesicles (2-5 mm) coalescing into bullae on an erythematous base with a hemorrhagic crust and minimal scale located on the lateral face, neck, chest, shoulder, back, and preauricular region spanning the V3-C5 dermatomes, without crossing midline. There was no oral or ocular involvement.

Laboratory evaluation revealed a positive VZV polymerase chain reaction (PCR) from both the lesion and serum, confirming active infection. VZV IgG titer was positive. The complete metabolic panel showed transaminitis with ALT 483 U/l (reference range 0-33 U/l), AST 214 U/l (reference range 0-32 U/l), and alkaline phosphatase of 242 U/l (reference range 45-87 U/l). Complete blood count revealed mild thrombocytopenia (platelet count 138 × 10^9^/l, reference range: 150-450 × 10^9^/l) with normal leukocyte and hemoglobin levels. Immunologic workup demonstrated elevated IgM (375 mg/dl, reference range: 40-230 mg/dl), normal IgG, modestly elevated CD3 and CD8 counts, slightly reduced CD4 count, and normal B and NK cell populations. Hepatitis serologies demonstrated nonreactive hepatitis B surface antigen (HbsAg), hepatitis B surface antibody (Anti-HBs), and hepatitis B core antibody (anti-HBc) as well as negative hepatitis A IgM antibody (IgM anti-HAV) indicating no evidence of prior hepatitis B infection or immunity despite the patient receiving both hepatitis A and B immunizations. The patient reported no sexual history or history of sexually transmitted infections. HIV serology obtained during hospitalization was negative.

She was managed with intravenous (IV) acyclovir 600 mg every 8 hours, morphine, gabapentin, and prednisone. Over the next several days, the rash extended to dermatomes V2-C6 and eventually crossed midline at C2-C3. The patient’s pain was severe, with marked allodynia and restricted range of motion of the neck and upper extremities. Infectious disease, dermatology, otolaryngology, allergy/immunology, pain medicine, and ophthalmology were consulted. Given the concern for potential clinical acyclovir resistance due to worsening of rash and the development of new lesions, therapy was changed to IV foscarnet (3552 mg or 60 mg/kg every 8 hours), cefazolin, and ketorolac. Following moderate clinical improvement, foscarnet was discontinued due to its potential for renal toxicity and requirement for intravenous administration, and the patient was transitioned to valacyclovir 1000 mg 3 times daily for 7 days per os (PO). The rash progressed with clinical features concerning for secondary superficial bacterial cellulitis. Wound cultures were not obtained; blood cultures remained negative. Treatment with IV cefazolin 2000 mg every 8 hours for 2 days followed by PO cephalexin 500 mg 3 times daily for 3 days led to resolution of the cellulitis.

She was discharged on oral valacyclovir after a 9-day hospital stay. The patient’s hearing loss, dermatologic lesions, and transaminitis resolved by the end of her hospital stay, with repeat laboratory testing demonstrating normalization of transaminases. Her facial droop also resolved on repeat neurologic examination. However, she continued to experience moderate neuropathic pain at the time of discharge and was referred for outpatient pain management.

Two weeks later, she was readmitted for recurrence of rash and worsening pain involving dermatomes C2-C6 and the occiput. Morphine was administered via patient-controlled analgesia (PCA) for pain control. Given the recurrence of lesions on valacyclovir therapy, there was a concern for potential clinical valacyclovir resistance—though resistance testing was not performed. As a result, antiviral therapy was escalated to IV foscarnet 3552 mg (60 mg/kg) and IV acyclovir 600 mg every 8 hours to provide broader antiviral coverage while awaiting clinical response. The patient continued to develop new lesions on this regimen and her pain progressed. Consequently, acyclovir was discontinued and a 7-day course of PO famciclovir 500 mg twice daily was initiated as an alternative antiviral with comparable efficacy against varicella-zoster virus. Her lesions resolved.

After 3 days on morphine PCA, she was weaned to PO immediate-release morphine 15 mg every 4 hours pro re nata (PRN) due to improved pain control. Four days later, this was transitioned to PO oxycodone 10 mg every 4 hours PRN and subsequently reduced to 5 mg every 4 hours PRN 2 days later. On the same day, the patient developed chills, nausea, fatigue, abdominal pain, intermittent diaphoresis, tremulousness, headache, and rhinorrhea. Given the recent opioid taper and transition from morphine PCA to lower-dose oral opioid therapy, the chronic pain team was concerned for possible opioid withdrawal. Management involved continuing her current oxycodone regimen with adjunctive ibuprofen and acetaminophen for symptomatic relief leading to clinical improvement. The patient’s dermatologic lesions resolved by the end of her second hospitalization (19 days duration), and she was discharged.

Genetic testing and primary immunodeficiency panel—including a 474-gene panel—were ordered through outpatient immunology and later returned negative for immunodeficiency or immune dysregulation. The patient also demonstrated a normal vaccine response to tetanus. Pneumococcal serologic testing showed protective antibody levels to 14 of 23 serotypes tested (61% response). While a normal response is typically defined as ⩾70% of serotypes 4 to 6 weeks following vaccination, the patient received the pneumococcal vaccination in infancy and her response was considered adequate.

Despite resolution of her cutaneous lesions, she developed postherpetic neuralgia characterized by a tingling sensation and persistent, sharp pain on the neck, scalp, and ear, which required ongoing management through the chronic pain medicine clinic. Her postherpetic neuralgia caused significant functional impairment, preventing her from completing the academic school year.

## Discussion

This case is notable for disseminated varicella-zoster virus infection with concomitant Ramsay Hunt Syndrome in an immunocompetent, fully immunized 17-year-old, confirmed by PCR testing of both serum and the vesicular lesion.

HZ typically occurs in adults as cell-mediated immunity declines with age.^5^ The incidence increases significantly after age 50 years, with rates of 3 to 5 cases per 1000 person-years in the general adult population, rising to 6 to 8 per 1000 at age 60, and 8 to 12 per 1000 at age 80.^6^ The introduction of the live attenuated zoster vaccine in 2006 and the recombinant subunit vaccine in 2017 has led to a measurable decline in incidence.^7^

HZ is uncommon in children, particularly among those who are immunocompetent and vaccinated.^8^ Between 2003 and 2014, the incidence of HZ in pediatric patients less than 18 years old was 74 per 100 000 persons, with immunized children showing a 78% reduction in risk compared to unvaccinated children.^9^ A 2023 systematic review identified only 16 studies reporting pediatric HZ incidence worldwide, underscoring the rarity of this diagnosis.^10^ Most cases occur in children older than 5 years, with the highest risk seen in those exposed to VZV in utero or during early infancy.^11^ Pediatric HZ classically presents with a dermatomal vesicular rash that is pruritic rather than painful, in contrast to adults in whom neuropathic pain predominates.^11^ Immunosuppressed children and those with chronic medical conditions demonstrate a higher risk of both infection and complications.^11^

Several reports have described severe or atypical manifestations of HZ in immunocompetent pediatric patients. Examples include a 14-year-old boy who developed laryngeal zoster with vagus neuropathy,^12^ an 11-year-old girl with HZ complicated by viral meningitis,^13^ and a 7-year-old boy with herpes zoster multiplex bilateris involving multiple noncontiguous dermatomes.^14^ Other cases describe HZ ophthalmicus children as young as five years old,^15,16^ and vaccine-associated zoster within a few years of immunization.^17,18^

Like these cases, our patient was immunocompetent but developed a severe, disseminated presentation. She was exposed to primary varicella at 6 months old, a known risk factor for HZ.^11^ However, unlike previously reported pediatric cases, her disease was associated with Ramsay Hunt Syndrome and hepatic involvement. Furthermore, her rash was acutely painful, contrasting the typically mild or painless course described in the pediatric population.

While HZ is generally mild in immunocompetent children, several complications can occur. In adults, these include postherpetic neuralgia, bacterial superinfection, ocular involvement (such as uveitis and keratitis), motor neuropathies, meningitis, and herpes zoster oticus.^19,20^ Pediatric complications are not well characterized in the literature but have included bacterial skin infections and, rarely, PHN—particularly in cases involving cranial dermatomes.^20^

Our patient’s course was complicated by superimposed bacterial cellulitis, opioid withdrawal during inpatient pain management, and persistent PHN requiring long-term pain therapy. Her acute opioid withdrawal was likely due to the opioid taper and transition from morphine PCA to lower-dose oral opioid therapy. It was managed by continuation of her opioid regimen with additional analgesics for symptomatic relief. Her PHN was most likely explained by the location of the vesicular lesions in the cranial dermatomes.^20^ Despite extensive immunologic evaluation, including a negative outpatient genetic immunodeficiency panel, no underlying immune abnormality was identified. Her significant functional impairment and inability to complete the academic school year highlight the profound physical and psychosocial impact of severe zoster in adolescents.

There are several limitations to this case report. One limitation is the reliance on patient-reported history, which may be subject to recall bias or incomplete recollection. For example, the patient was unable to remember the initial dose of oral valacyclovir prescribed at the outside hospital and records were unavailable for our review. However, the remainder of the patient’s data was collected via the electronic medical record. Another limitation is lack of follow-up after discharge. As a result, we were unable to provide a comprehensive report of her clinical recovery following treatment. Finally, this case reports a single clinical observation which restricts generalizability to broader populations.

## Conclusion

This case illustrates a rare, but severe presentation of disseminated herpes zoster with visceral involvement and Ramsay Hunt Syndrome in an otherwise healthy adolescent. Children who contract VZV during their first year of life are more likely to develop herpes zoster in childhood. Although severe HZ is uncommon in immunocompetent, vaccinated patients, clinicians should maintain a high level of suspicion in pediatric patients who present with rash and neuropathic symptoms, as these cases may lead to significant morbidity. Unusually painful, extensive, or neurologically complex HZ infections in children warrant prompt evaluation. Timely virologic testing, antiviral therapy, and proactive pain management are critical to improving clinical outcomes. Despite appropriate management, complications such as postherpetic neuralgia, bacterial superinfection, and meningitis may still occur. Early recognition and coordinated multidisciplinary care are essential to reducing long-term neurologic complications and optimizing pain management, ultimately improving recovery in patients with herpes zoster.
